# Diet and traffic: anthropogenic factors that influence stress-related hormone levels in African clawless otters

**DOI:** 10.1093/conphys/coaf087

**Published:** 2025-12-13

**Authors:** Marli Burger, Andre Ganswindt, Andrea B Webster, Juan Scheun, Tshepiso L Majelantle

**Affiliations:** Mammal Research Institute, Faculty of Natural and Agricultural Sciences, University of Pretoria, University Rd, Hatfield, Pretoria, Private bag X20, 0028, South Africa; Mammal Research Institute, Faculty of Natural and Agricultural Sciences, University of Pretoria, University Rd, Hatfield, Pretoria, Private bag X20, 0028, South Africa; Mammal Research Institute, Faculty of Natural and Agricultural Sciences, University of Pretoria, University Rd, Hatfield, Pretoria, Private bag X20, 0028, South Africa; Department Nature Conservation, Tshwane University of Technology, Staatsartillerie Road, Pretoria West, Pretoria, Private Bag X680, 0001, South Africa; Mammal Research Institute, Faculty of Natural and Agricultural Sciences, University of Pretoria, University Rd, Hatfield, Pretoria, Private bag X20, 0028, South Africa; Brain Function Research Group, Department of Physiology, University of Witwatersrand, 7 York Road, Parktown, Johannesburg, Private Bag 3, 2050, South Africa

**Keywords:** African clawless otter, anthropogenic disturbance, diet, faecal glucocorticoid metabolites, stress, transformed habitat

## Abstract

Environmental and anthropogenic factors significantly drive adrenocortical activity of animals, affecting their behaviour, distribution and survival. Understanding how animals respond to such drivers is essential for effective conservation. Spraint samples from free-ranging African clawless otters (*Aonyx capensis*) and camera trap data were collected from study sites categorized as natural or artificially transformed based on differences in anthropogenic disturbance levels. To determine if there were significant differences in faecal glucocorticoid metabolite (fGCM) concentrations between the Natural (Kalkfontein Nature Reserve) and Transformed (Millstream Farm) sites, we ran a linear model that included sex, season, habitat type and their interaction. fGCM concentrations differed significantly between the sexes (df = 1; *F*_1,106_ = 11.180; *P* = 0.001); with males (*n* = 32; 0.608 ± 0.367 μg/g DW) having significantly higher fGCM concentrations compared to females (*n* = 79; 0.414 ± 0.399 μg/g DW, *P* = 0.006). The fGCM concentrations differed significantly between seasons (df = 1; *F*_1,106_ = 45.268; *P* < 0.001), with those in the dry winter season significantly higher (*n* = 66; 0.631 ± 0.420 μg/g DW), compared to the wet summer season (*n* = 45; 0.234 ± 0.199 μg/g DW). The fGCM concentrations differed significantly between habitat type (df = 1; *F*_1,106_ = 6.026; *P* = 0.016) with fGCM concentrations of individuals from the KNR natural site (*n* = 34; 0.285 ± 0.199 μg/g DW) being significantly lower compared to those measured in individuals at the MF transformed site (*n* = 77; 0.552 ± 0.436 μg/g DW). Finally, the difference in fGCM concentrations between locations however were not dependent on season (df = 1; *F*_1,106_ = 0.369; *P* = 0.544). Anthropogenic disturbance and alterations to the natural and varied prey-base of African clawless otters in an anthropogenically transformed site significantly affect their adrenocortical activity. Future research should focus on how these animals respond to anthropogenic disturbance, and what effects disturbance has on their behaviour, distribution and fitness. Mitigating human–otter conflict requires incorporating such behavioural responses into management strategies.

## Abbreviations

BSA: bovine serum albumin
CMO: carboxymethyloxime
DADOO-biotin: N-biotinyl-1,8-diamino-3,6-dioxaoctane
Dw: dry weight
fGCMs: Faecal glucocorticoid metabolites
GC: Glucocorticoid
HS: hemisuccinate
KNR: Kalkfontein Dam Nature Reserve
MF: Millstream farm

## Introduction

The human population is close to 8 billion people and continues to grow at a rate of approximately 0.71% per year (Gu *et al.,* 2021). Our increasing demands for food, water, land and energy resources results in increasing anthropogenic activities associated with agriculture, urbanization and industrial activities (Huang *et al.,* 2010). These activities are generally accompanied by habitat conversion or destruction, fragmentation, overexploitation and pollution, which transform and reduce natural habitats and ecosystems (Huang *et al.,* 2010; Mahmoud and Gan, 2018). As a result, humans and their associated anthropogenic activities are the main cause of global biodiversity loss (Riley *et al.,* 2003; Wingfield, 2013) and up to 50% of the world’s bird and mammal species are expected to suffer extinction in the next two to three centuries (Prakash and Verma, 2022). The consequences of environmental change on wildlife include changes in their diet, behaviour, distribution, physiology, growth, fitness and immune response. Alone or in combination, these factors impact survival (Halfwerk and Slabbekoorn, 2015; Harding *et al.,* 2019).

The nutritional requirements of an animal’s diet are linked to sex and different life history traits, such as growth and development, fitness, health and life expectancy (Engebretson, 2006; Pellizzon, 2016; Yuan *et al.,* 2018). In this regard, a diverse diet, including high-quality prey items is essential for animal survival and wellbeing (Pellizzon, 2016; Yuan *et al.,* 2018). The rapid transformation of natural landscapes and ecosystems causes alterations of ecological food webs, which can inhibit an individual’s ability to meet the full spectrum of nutrients necessary for well-being or even survival (Grant and Albright, 2000; Birnie-Gauvin *et al.,* 2017). Such dietary imbalances are likely to affect a number of processes including animal growth, reproduction, behavioural parameters, physiological functions (including blood parameters) and adult survival rates (Catanese *et al.,* 2013;Margalida *et al.,* 2014; Spaan *et al.,* 2020). Nutritional stress can also lead to migration of individuals; however, in cases where migration is not possible, animals may be trapped in unsuitable environments (Margalida *et al.,* 2014; Spaan *et al.,* 2020). The additive effects of continuous human-mediated and seasonal declines in food availability, abundance and quality can negatively affect species stress responses, which has implications for survival and welfare.

In a biological context, stress can be defined as a general syndrome resulting from a perceived stimulus (stressor) that threatens or appears to threaten the homeostasis of an individual (Selye, 1936). Stressors can include anthropogenic disturbance (Majelantle *et al.,* 2020), sex-related life history traits (Crossey *et al.,* 2018; Crossey *et al.,* 2020), diet (Nemeth *et al.,* 2021; Bragg *et al.,* 2024) and disease status such as parasitic load (O’Dwyer *et al.,* 2020). Once a stressor has been recognized, animals initiate behavioural, autonomic and neuroendocrine responses to restore homeostasis (Moberg, 2000). The endocrine stress response activates the sympatho-adrenal medullary system and hypothalamic–pituitary–adrenal (HPA) axis. Activation triggers physiological and behavioural responses including increased catecholamine and glucocorticoid (GC) secretion over a period of minutes, hours or days to help individuals cope with threats (Henry, 1991; Wilson *et al.,* 2016; Moberg, 2000; Sapolsky, 2002). These elevated hormone concentrations lead to increased glucose concentrations, cardiac output, cognitive function and muscle energy diversion (Sapolsky *et al.,* 2000; Wingfield and Romero, 2001; Wielebnowski, 2003) and decreased reproductive and gastrointestinal functions (Mӧstl and Palme, 2002; Palme, 2019). Acute stress enhances overall fitness and survival (Mӧstl and Palme, 2002; Wielebnowski, 2003), while chronic or prolonged GC elevations can suppress the immunity, hinder growth and reproduction and cause neurodegeneration and tissue atrophy (Sapolsky, 1992; Sapolsky *et al.,* 2000; Mӧstl and Palme, 2002). Responses to stressors are often species- and sex-specific, indicating differences in life history traits, physiology and environmental context (Sapolsky *et al.,* 2000). Monitoring GC patterns in animals can provide insights into their ability to cope with relevant environmental and physiological stressors and can therefore be used to assess animal welfare in captive and free-ranging individuals. Hormones have traditionally been quantified in blood; however, steroid hormone concentrations can also be non-invasively measured via respective hormone metabolites in matrices such as faeces. Faecal glucocorticoid metabolites (fGCM) are less affected by either circadian rhythms or pulsatile secretory patterns (Monfort *et al.,* 1993) and are therefore often used as a reliable indicator of stress experienced by an individual (Palme *et al.,* 2005; Sheriff *et al.,* 2011; Möstl, 2014).

Otters are primarily piscivorous top predators of aquatic habitats (Toweill, 1974; Bowyer *et al.,* 2003). The distribution of African clawless otters extends throughout Africa from Senegal and Mali in West Africa through to Ethiopia and Sudan in the northeastern parts of Africa (Somers and Nel, 2013; Jacques *et al.,* 2015). With the exclusion of the Congo basin, their range further stretches throughout the eastern parts of Africa southwards to the southernmost tip of Africa, in the Western Cape. These otters occupy a diverse range of freshwater habitats, including rivers, marshes, dams and lakes, as well as certain coastal intertidal zones provided access to fresh water sources is also available (Somers and Nel, 2004; Somers and Nel, 2013; Jacques *et al.,* 2015). In addition, these animals occur in urban/transformed and natural landscapes. African clawless otters occurring in transformed landscapes occurred in higher densities but had higher fGCM concentrations compared to otters in natural landscapes (Majelantle 2020, 2021). However, the study was limited as sex, diet and anthropogenic linked parameters were not investigated. Thus, the aim of this study was to examine the effect of environmental and anthropogenic disturbance on fGCM concentrations in African clawless otters to evaluate how those relationships vary by sex, anthropogenic disturbance, diet and season.

## Materials and Methods

This study was conducted with the approval of the University of Pretoria’s Research and Animals Ethics and Care Committees (NAS087/2020) and complies with the Department of Agriculture, Land Reform and Rural Development (DALRRD) Section 20 requirements for animal diseases (SDAH-Epi-21 110 813 130).

### Study sites and sample collection

Spraint samples from free-ranging African clawless otters and camera trap data were collected from study sites categorized as natural or transformed based on initial visual assessments and on differences in food availability and quality, water quality and anthropogenic disturbance levels.

**Figure 1:**
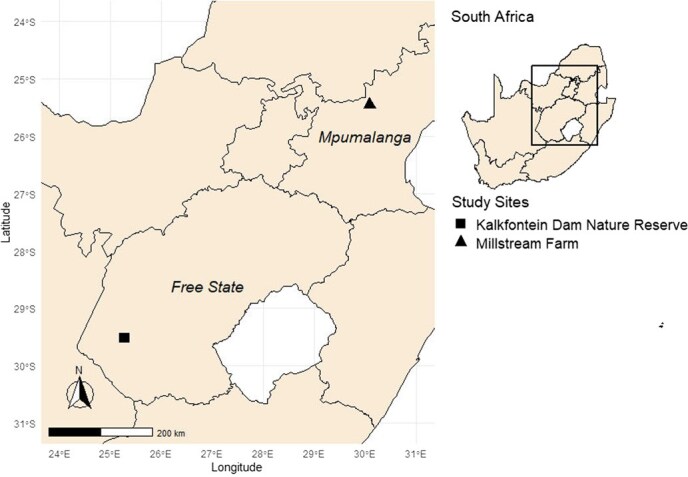
Representation of spatial locations where African clawless otter fresh faecal samples were collected during the wet season (3 November 2021–12 January 2022) and dry season (5 July–30 July 2021)

Kalkfontein Dam Nature Reserve (KNR); the natural site is situated in the Free State province (29°31′10.056″S, 25°16′48.468″E; Fig. 1) of South Africa. Kalkfontein Dam has a capacity of 258 274×10^3^ m^3^ and was built along the Riet River to supply irrigation water to surrounding commercial crop farms. The reserve supports a healthy aquatic system with a variety of birds, plants and fish species (DESTEA, 2025). Otters therefore have access to high quality, quantity and variety of dietary items and anthropogenic disturbance is limited to daily or weekend anglers and holidaymakers. Spraint samples were collected from five previously identified latrines around the Kalkfontein Dam during the dry winter season (5 July–11 July 2021; *n* = 34) and four latrines during the wet summer season (5 January–12 January 2022; *n* = 19). The average rainfall during the winter study period was 0 mm and temperatures ranged between −8 and 22°C whereas the average rainfall during the summer study period was 7 mm and temperatures ranged between 13 and 33°C.

Millstream Farm (MF); the transformed site is situated in Mpumalanga province (25°27′7.272″S, 30°5′30.732″E; Fig. 1), South Africa. The farm covers 408 ha and has a series of natural and artificially constructed dams, weirs, ponds and lakes and is built along the Witpoort River. Water bodies around the farm are used for the commercial farming of rainbow trout (*Oncorhynchus mykiss*), which supports fly-fishing for recreational purposes. Farmed trout in this system are supplied with supplements and are the primary prey species available to resident otters. Millstream farm is home to a variety of bird, mammal, fish and reptile species, which are exposed to high volumes of holiday makers, visitors and alterations to their natural and varied prey-base. Spraint samples were collected from five latrines around Millstream during the dry winter season (22 July–30 July 2021; *n* = 65) and from six latrines during the wet summer season (3 November–9 November 2021; *n* = 39). The average rainfall during the winter study period was 0 mm and temperatures ranged between −5 and 21°C whereas the average rainfall during the summer study period was 2 mm and the temperatures ranged between 2 and 32°C.

### Camera trap data

To assess anthropogenic disturbance, Browning Infra-red Recon Force Elite HP4 (22MP) trail cameras (subsequently referred to as camera traps) were used to determine human presence and activity levels. Camera traps were mounted to sturdy trees or posts, placed 1-2 m from the road at a height of 0.5–1.5 m above ground and angled downwards to avoid sun interference along routes leading towards water bodies where otter activity had previously been observed (Duke and Quinn, 2008; Fairfax *et al.,* 2014; Miller *et al.,* 2017). Vegetation in front of the camera lens was cleared to ensure visibility and prevent false triggers (Fairfax *et al.,* 2014). Camera traps, programmed for long-range detection (up to ±24 m), captured 3 photographs at 3-s intervals. Cyclists and pedestrian data were grouped together, while cars were categorized separately.

### Faecal sample collection and processing

#### Sample identification and collection

African clawless otter spraints were primarily identified based on physical characteristics including size (diameter = ca. 25 mm), shape, characteristic smell and tracks surrounding latrines (Liebenberg, 1990; Rowe-Rowe, 1997). On some occasions, spraints were collected after otters were seen defaecating in the area; however, most samples were collected from previously identified latrine sites using anal jelly texture, temperature and moisture content as indicators of freshness. Given the use of latrines for olfactory communication between individuals, only a fragment of each faecal deposit was collected to minimize communication disturbance. In addition, deposits were homogenized *in situ* using latex gloves and subsamples were collected from the centre of the faecal pile to avoid contamination by substrate. Thereafter, samples were placed in individually labelled plastic screw top containers and kept cool in a portable cooler box with ice bricks and frozen within three hours. The remaining faecal samples were disfigured by flattening to avoid the collection of old samples. All latrines were visited daily, between 06:00 and 09:00. If any uncertainty existed related to sample age, samples were not collected. A total of 157 spraint samples were collected from 20 different free-ranging otter latrines. During the winter period, 99 samples were collected from 10 latrines, while 58 samples were collected from 10 latrines during the summer period. Samples were kept frozen and transported by road to the University of Pretoria’s Endocrine Research Laboratory, where they were stored frozen at −20°C until further processing and analysis (Ganswindt *et al.,* 2002).

#### Analysis for diet and parasites

Spraint samples were used to classify dietary differences of otters at each study site. Samples were lyophilized and the content visually inspected and sorted into one of ten categories: crabs, fish, mammals, insects, amphibians, reptiles, birds, molluscs, unidentifiable material and non-food items (Somers and Purves, 1996). Vertebrates (fish, mammals, amphibians, reptiles and birds) were identified by means of skeletal remains together with distinctive keratin structures such as scales, hair, feathers, beaks, nails, talons or claws and on occasion undigested skin. Exoskeletons were used to identify invertebrates (crabs, insects and molluscs; Somers and Purves, 1996). Most of the remains could be identified with the naked eye, but prey items such as insect remains were classified using a Vickers light microscope at ×4/0.10 or 10/0.25 magnification depending on the size of the item. To identify important food items and determine the most dominant prey item at each site, we calculated the relative percentage of occurrence (RPO) using the following equation (Erlinge, 1968). See Burger et al. (2025) for detailed results:


\begin{equation*} \mathrm{RPO}=\frac{\begin{array}{c}\mathrm{Total}\ \mathrm{number}\ \mathrm{of}\ \mathrm{occurrences}\ \mathrm{of}\ \mathrm{a}\ \mathrm{s}\mathrm{pecific}\\ \mathrm{prey}\ \mathrm{item}\ \mathrm{in}\ \mathrm{a}\mathrm{ll}\ \mathrm{of}\ \mathrm{the}\ \mathrm{faecal}\ \mathrm{samples}\end{array}}{\begin{array}{c}\mathrm{Total}\ \mathrm{number}\ \mathrm{of}\ \mathrm{occurrences}\ \mathrm{of}\ \mathrm{a}\mathrm{ll}\ \mathrm{prey}\\ \mathrm{item}\mathrm{s}\ \mathrm{recorded}\ \mathrm{in}\ \mathrm{a}\mathrm{ll}\ \mathrm{of}\ \mathrm{the}\ \mathrm{faecal}\ \mathrm{samples}\end{array}}\times100 \end{equation*}

The RPO represents the percentage of a specific prey item in relation to the total number of prey items consumed by the population (Rowe-Rowe and Somers, 1998; Crowley *et al.,* 2018). The most dominant prey item was determined based on the prey item with the highest RPO.

During the sorting and categorization of food items, faecal samples were visually inspected for the presence or absence of parasites (Fig. S1 for reference of faecal samples with parasites present). Considering that parasites were only detected at one study site, the dataset did not allow detailed exploration of parasite identification or load and was thus beyond the scope of this study.

#### Faecal sample extraction and fGCM quantification

Following diet analysis and visual inspection for parasite presence, spraint samples (*n* = 157) were pulverized and sieved to remove debris and undigested particulate matter. Thereafter, faecal powder (0.10–0.11 g) from each sample was measured and 3 ml of 80% ethanol in water was added. Contents were vortexed for 15 min, centrifuged at 1500 g for 10 min and the supernatant (here on referred to as faecal extracts) decanted into sealed Eppendorf tubes and stored at −20 until analysis (Ganswindt *et al.,* 2002).

Faecal extracts were analyzed for faecal glucocorticoid (fGCM), faecal progestagen (fPM) and faecal androgen (fAM) metabolite concentrations using a cortisol enzyme immunoassay (EIA; Palme and Möstl, 1997), Progesterone EIA (Schwarzenberger *et al.,* 1996) and Epiandrosterone EIA (Palme and Möstl, 1993), respectively. These EIAs were previously established for quantifying fGCMs (Majelantle *et al.,* 2020), fPMS and fAMs (Burger *et al.,* 2024) in African clawless otters. All faecal extracts were measured in accordance with procedures outlined by Ganswindt et al. (2002). Details on EIA sensitivities, antibodies, labels, standards and intra- and inter-assay coefficients of variation of high- and low-quality controls are provided in supplementary material (Table S1). Serial dilutions of faecal extracts resulted in displacement curves parallel to the respective standard curves and had variation slopes of respective trend lines of <5% for all three EIAs. Steroid concentrations are expressed per mass of dry faecal matter. All faecal steroid extractions and analyses were conducted at the University of Pretoria’s Endocrine Research Laboratory, South Africa.

### Data analysis

Following the methods described by Burger et al. (2024), faecal samples were assigned sex using fAM and fPM ratios. To determine if there were significant differences in faecal glucocorticoid metabolite concentrations across the two habitat types, natural (KNR) and transformed (MF), a linear model that included sex, season, habitat type and their interaction was run. Data for habitat types (natural and transformed) could not be included in a model in combination with environmental, anthropogenic and diet factors as some factors were unique to location thus violating the assumption of homogeneity of linear models. Therefore, the dataset was separated according to habitat type.

For each habitat type, the effect of sex, season, anthropogenic factors (number of cars) and diet (most dominant prey item; Burger *et al.,* 2025) on fGCM concentrations were evaluated using a linear model. In addition, the presence/absence of parasites in faeces was included in the model for the transformed site, as only this site had parasites in faecal samples.

For all models, normality was assessed by testing the model residuals using the Shapiro–Wilk test, Levene’s test and visually using quantile plots. The fGCM concentrations and residuals for all models followed a positively skewed distribution, necessitating log_10_ transformation prior to statistical analysis. All statistical analysis were conducted using R (R Core Team, 2022), and statistical significance was determined at *P* < 0.05. All concentrations are presented as mean ± standard deviation (SD) μg/g dry weight (DW).

## Results

**Figure 2:**
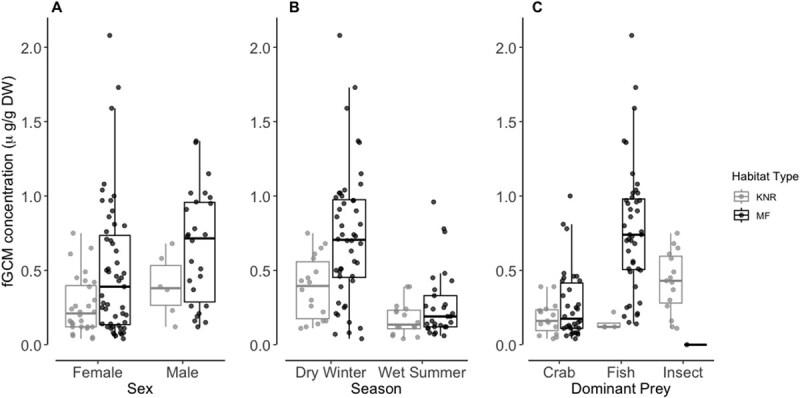
Box plot of fGCM concentrations (*y*-axis; median and inter-quartile range) of otters across the different study sites (*x*-axis; (A) Sex, (B) Season and (C) Dominant Prey item) in KNR (natural, grey) and the MF (transformed, black).

Using the results from Burger et al. (2024), sex could successfully be assigned to 111 of the 157 collected samples (79 from females and 32 from males). Consequently, the remaining 46 samples assigned to unknown sex were excluded from further analysis. The model including sex, season, habitat type and their interaction was a significant predictor of fGCM concentrations (*F*_4,106_ = 17.370; *P* < 0.001). There was a significant difference in fGCM concentrations between the sexes (df = 1; *F*_1,106_ = 11.180; *P* = 0.001; Fig. 2A); where males (*n* = 32; 0.608 ± 0.367 μg/g DW) had significantly higher fGCM concentrations compared to females (*n* = 79; 0.414 ± 0.399 μg/g DW, *P* = 0.006). The fGCM concentrations differed significantly between seasons (df = 1; *F*_1,106_ = 45.268; *P* < 0.001; Fig. 2B), with those in the dry winter season significantly higher (*n* = 66; 0.631 ± 0.420 μg/g DW), compared to the wet summer season (*n* = 45; 0.234 ± 0.199 μg/g DW). The fGCM concentrations differed significantly across habitat type (df = 1; *F*_1,106_ = 6.026; *P* = 0.016; Fig. 2) with fGCM concentrations of individuals from the KNR natural area (*n* = 34; 0.285 ± 0.199 μg/g DW) being significantly lower compared to those measured in individuals at the MF transformed site (*n* = 77; 0.552 ± 0.436 μg/g DW). Finally, the difference in fGCM concentrations between locations however was not dependent on season (df = 1; *F*_1,106_ = 0.369; *P* = 0.544).

### Kalkfontein nature reserve (natural site)

The model including sex, season, dominant prey item and previous day’s number of cars was a significant predictor of fGCM concentrations in samples collected from KNR (*n* = 34, *F*_5,28_ = 5.748; *P* = 0.001). There was no significant difference (*F*_1,28_ = 0.081; *P* = 0.778; Fig. 2A) in fGCM concentrations from faecal samples collected from females (*n* = 28; 0.261 ± 0.192 μg/g DW) and males (*n* = 6; 0.395 ± 0.209 μg/g DW) at KNR. Likely due to the low sample size from males. Likewise, there was no significant difference (*F*_1,28_ = 2.097; *P* = 0.159; Fig. 2B) between fGCM concentrations from samples collected during the dry winter season (*n* = 18; 0.383 ± 0.211 μg/g DW) and the wet summer season (*n* = 16; 0.174 ± 0.110 μg/g DW). In addition, there was no significant difference (*F*_1,28_ = 3.058; *P* = 0.063; Fig. 2C) in fGCM concentrations between samples where the dominant food items were crab (*n* = 15; 0.181 ± 0.111 μg/g DW), fish (*n* = 4; 0.145 ± 0.050 μg/g DW) and insects (*n* = 15; 0.425 ± 0.206 μg/g DW). Finally, there was a significant, but weak negative relationship between the fGCM concentrations and previous days number of cars (*F*_1,28_ = 4.602; β = −0.108; *R*^2^ = 0.13; *P* = 0.041).

### Millstream farm (transformed site)

The model for sex, season, dominant prey item, presence of parasites and previous days’ number of cars was a significant predictor of fGCM concentrations in samples collected from MF (*n* = 77, *F*_5,71_ = 18.740; *P* < 0.001). There was a significant difference in fGCM concentrations between the sexes (*F*_1, 71_ = 9.390; *P* = 0.003; Fig. 2A) with male fGCM concentrations (*n* = 26; 0.658 ± 0.381 μg/g DW) being significantly higher than females (*n* = 51; 0.498 ± 0.456 μg/g DW). However, there was no significant difference (*F*_1,71_ = 3.438; *P* = 0.069 Fig. 2B) between fGCM concentrations from samples collected during the dry winter season (*n* = 48; 0.724 ± 0.443 μg/g DW) and the wet summer season (*n* = 29; 0.268 ± 0.229 μg/g DW). Otters with a diet dominated by fish (*n* = 43; 0.775 ± 0.431 μg/g DW) had significantly higher fGCM concentrations compared to otters with a crab-dominated diet (*n* = 34; 0.270 ± 0.234 μg/g DW; *F*_1,71_ = 20.217; *P* < 0.001; Fig. 2C). Interestingly, there was no significant difference (*F*_1,71_ = 0.238; *P* = 0.627) in fGCM concentrations between otters with parasites (*n* = 14; 0.656 ± 0.438 μg/g DW) and otters without parasites (*n* = 63; 0.529 ± 0.436 μg/g DW) in their faeces. However, this is likely due to the low sample size from samples, which had parasites present. Although weak, there was a significant positive relationship between the fGCM concentrations and the previous day’s number of cars (*F*_1, 71_ = 16.696; β = 0.077; *R*^2^ = 0.08; *P* < 0.001).

## Discussion

Landscapes transformed by anthropogenic presence and activity can influence wildlife in multiple ways. Here, we assessed how environmental and anthropogenic factors (different habitat types, sex, season, number of cars and diet) drive adrenocortical activity in African clawless otters. Our results suggest that there are sex-associated differences in African clawless otter stress-related hormone concentrations. In addition, our findings are similar to Majelantle et al. (2020), which show otters occurring at the transformed site (Millstream Farm), had significantly higher fGCM concentrations compared to the natural site, (Kalkfontein Nature Reserve). The results suggest the main driver of this difference is potentially due to diet as it was a significant predictor of fGCM concentrations at the transformed site.

Results from this study suggest that male otters had considerably higher fGCM concentrations overall compared to their female counterparts. Sex-related variation in fGCM concentrations in response to stressors are commonly observed in endocrine studies (see Touma *et al.,* 2003; Webster *et al.,* 2018; Rudolph *et al.,* 2020; O’Brien *et al.,* 2022). Hormone metabolism, the excretion of specific metabolites within faeces and alterations in faecal steroid metabolites post-defaecation can be sex-specific (Touma *et al.,* 2003; Palme *et al.,* 2005; Crossey *et al.,* 2018). These differences have been ascribed to sex-specific differences in life history traits (Rudolph *et al.,* 2020) as well as variation in hormone metabolism and excretion (Touma *et al.,* 2003). A study conducted by Girard-Buttoz et al. (2014) found that male long-tailed macaques (*Macaca fascicularis*) had higher fGCM concentrations during the breeding season as a result of mate-guarding, increased vigilance time and increased aggression towards other males. It is possible that male African clawless otters have elevated fGCM concentrations due to the consequences associated with breeding (Girard-Buttoz *et al.,* 2014). Alternatively, the sex-related differences observed in fGCM concentrations may be linked to sex-specific differences in HPA activity in which the HPA axis response may differ with regards to diet and energy demands (or other environmental factors) between sexes (Parra-Montes de Oca *et al.,* 2021). However, given the knowledge gaps and inconsistencies related to African clawless otter reproductive biology (Larivière, 2001; Somers and Nel, 2013), it is challenging to determine the breeding season of African clawless otters.

A comparison of fGCM concentrations between study sites suggests that otters in the transformed site (MF) had significantly higher fGCM concentrations compared to those at the natural site (KNR). These results align with Majelantle et al. (2020), suggesting increased adrenocortical activity in response to site-specific drivers. In addition, fGCM concentrations of otters at both study sites investigated here were higher during the dry winter season, compared to the wet summer season, suggesting site-specific drivers of adrenocortical activity in otters are linked to the different seasons. The number of cars at the natural site was the only significant, although weak, driver of adrenocortical activity at the natural site. Lower fGCM concentrations in response to anthropogenic presence could be an indication of habituation. A study conducted by Scheijen et al. (2021), found that giraffes (*Giraffa camelopardalis*) had lower fGCM concentrations in response to anthropogenic presence once habituated. Considering the weak relationship between fGCM concentrations and the previous day’s number of cars, it is likely that additional data and analysis is required to make conclusive inferences related to anthropogenic presence at this site, and this result should be interpreted with caution.

Investigation of African clawless otter diet (Burger *et al.,* 2025) suggests that dominant prey item consumed was a significant driver of glucocorticoid secretion at the transformed site. This finding suggests that individuals who fed predominantly on fish during the study period at this site had significantly higher fGCM concentrations compared to individuals that fed primarily on crab. Although there was no significant relationship between otters with or without parasites in the faeces, it should be noted that subsamples of scat were collected to facilitate olfactory communication between conspecifics, which creates the possibility of false negatives in the dataset. As a result, the elevated fGCM concentrations in individuals that ate predominantly fish at the transformed site could possibly be explained by the increased parasite load observed in samples containing fish compared to crab (M. Burger, pers. obs.). Endoparasites, including nematodes and trematodes, are commonly seen in African clawless otters (Round, 1968; Somers and Nel, 2013; Somers *et al.,* 2024), as well as numerous other otter species across the globe (Schiller, 1954; Kimber and Kollias, 2000; Sherrard-Smith *et al.,* 2009). According to Lutermann et al. (2014, 2015), the parasite burden observed in different species is often linked to specific diets or prey items consumed. Rainbow trouts are hosts or intermediate hosts for numerous parasites including trematodes, nematodes and acanthocephalans and in this regard are rather susceptible to parasitic infections globally (Torres, 1995; Buchmann and Bresciani, 1997; Jørgensen *et al.,* 2009). Farmed trout typically have a higher parasite load than wild trout due to increased stocking densities in aquaculture systems (Lafferty *et al.,* 2015; Krkošek, 2017). This would allow farmed trout to transmit parasites to aquatic predators in these systems.

Given that specific characterization of parasites was beyond the scope of this study, a catalogue of specific taxa was not compiled. However, parasites observed in the faeces (Fig. S1) showed some resemblance to threadworm (*Strongyloides* sp.) and duodenal hookworm (*Ancylostoma duodenale)*, which have previously been reported in African clawless otters (Somers *et al.,* 2024). Considering the lack of research related to parasitic diseases in African clawless otters, it is unknown whether these parasites are pathogenic parasites that may cause disease or mortality in otters. Globally, parasitic diseases and mortality, resulting from ingestion of parasites during the consumption of fish are common in various otter species (Schiller, 1954; Kimber and Kollias, 2000; Kreuder *et al.,* 2003). However, mortality and pathogenicity are dependent on the species of parasite and in some cases, the abundance of the parasites present (Kimber and Kollias, 2000). It should be noted that there were several unexplained otter mortalities at the transformed site prior to sample collection for this study with no clear sign of injuries or cause of death (Millstream Farm personnel pers. comm.). Thus, the possibility of these mortalities being related to parasitic diseases cannot be ruled out.

In a study conducted by Haliloğlu *et al*. (2002), the fatty acid content of muscle was compared in three different trout species (rainbow trout, brown trout; *Salmo trutta* and arctic charr; *Salvelinus alpinus*). All three species were raised under the same conditions and were fed the same diet with identical fatty acid composition. Their results showed that rainbow trout had significantly higher saturated fatty acid content compared to both brown trout and arctic charr, and significantly lower monounsaturated fatty acid content compared to the other two fish species. Saturated fatty acids are recognized obesogens that increase body fat and overall body weight of mammals (Takeuchi *et al.,* 1995; Bell *et al.,* 1997; Hariri and Thibault, 2010). High-fat diet-induced obesity is known to cause neuronal damage, low fertility and compromise reproductive success in mammals (Crean and Senior, 2019; Seo *et al.,* 2020). Obesity and high body mass indices caused by high-fat diets causes hyperresponsiveness of the HPA axis, resulting in elevated glucocorticoid production and secretion (Pasquali and Vicennati, 2000; Legendre and Harris, 2006; Incollingo Rodriguez *et al.,* 2015). Thus, the high fat content of a homogenous diet of fish at the transformed site could possibly be driving adrenocortical activities in otters. Future studies should investigate the body mass index and body condition of otters at this site to better understand if the high-fat trout diet is causing obesity in the species, thus driving glucocorticoid secretion. In addition, the trout at the transformed site are fed with 12.5 kg of Aqua-Plus Trout Feed supplements every month between May and September. These pellets contain min 300 protein, min 18 lysine, min 60 fat, max 100 moisture, max 30 crude protein, max 30 calcium, min 7 phosphorus (g/kg). While these are all standard components of fish feed, not inherently harmful to animals, there could be risks to bone health, overall growth, kidneys and blood chemistry related to increased dietary intake of e.g. phosphorus and calcium, which could explain the elevated fGCM concentrations (Chang and Anderson, 2017; Alagawany *et al.,* 2021; Ugrica *et al.,* 2021).

African clawless otters at the transformed site had higher fGCM concentrations when exposed to higher anthropogenic activity in the form of cars. Anthropogenic disturbance has been known to cause physiological changes in animals, including adrenocortical changes, such as elevations in glucocorticoid or cortisol concentrations (Tempel and Gutiérrez, 2004; Muehlenbein *et al.,* 2012; Piñeiro *et al.,* 2012). Anthropogenic presence includes direct contact with animals, close proximity to animals and anthropogenic noise (Martin and Réale, 2008). While some individuals or populations have become habituated to anthropogenic presence, others experience deleterious effects in response to anthropogenic presence and disturbance (Muehlenbein *et al.,* 2012; Villanueva *et al.,* 2012). Majelantle et al. (2020), found similar results when otters at Millstream farm, characterized by increased human presence, had considerably higher fGCM concentrations compared to those found in natural areas with low anthropogenic presence. The same author also reported that otters at Millstream Farm altered their activity periods from crepuscular to nocturnal, possibly to avoid periods of increased human activity and to limit their encounters with humans (Majelantle *et al.,* 2021). Webster et al. (2021) noticed a similar shift in activity patterns of mesocarnivores, including African clawless otters in response to human disturbance. Similarly, black bears (*Ursus americanus*) and African buffalo (*Syncerus caffer*) have also been reported to shift their activity periods from diurnal, to crepuscular or nocturnal in response to human activities during the day (Beckmann and Berger, 2003; Erena, 2022). This can lead to missed foraging opportunities resulting in nutritional stress, which can further increase the secretion of glucocorticoids (Tucker *et al.,* 2018; Erena, 2022).

Similarly, animals exhibit spatial avoidance by limiting their movement to avoid areas occupied by and associated with humans (Krüger *et al.,* 2015; Mancinelli *et al.,* 2018; Erena, 2022). These responses can lead to a reduction in animal home range and complete abandonment of territories (Krüger *et al.,* 2015; Tucker *et al.,* 2018), which can result in restricted food availability and insufficient availability of shelter and denning sites in the remaining habitat. According to Suraci et al. (2019), elevated physiological stress and accompanying behavioural changes in animals can partially be attributed to the fear of humans as predators. Human-otter conflict is a major problem in South Africa, especially in trout-stocked waters used for recreational fly-fishing purposes (Jacques *et al.,* 2015; de Vos, 2018). It is possible that otters may perceive humans as predators, even more so as human-otter conflict is suggested to continue and is somewhat increased in urban and peri-urban areas (Jordaan *et al.,* 2020). Chronically elevated glucocorticoids can lead to suppressed immune responses (Sapolsky *et al.,* 2000; Mӧstl and Palme, 2002), reduced growth and reproduction (Sapolsky *et al.,* 2000; von der Ohe and Servheen, 2002; Wielebnowski, 2003), neurodegeneration (Sapolsky, 1992), and atrophy of cells and tissue (Basu *et al.,* 2001; Mӧstl and Palme, 2002). In this regard, the prolonged elevation of glucocorticoids impedes the proper function of the stress response and decreases the efficiency with which an animal can respond to subsequent environmental and physiological stressors (Wingfield and Romero, 2001). In addition, Suraci et al. (2019) mention that elevated glucocorticoid concentrations in response to anthropogenic presence might have additional unknown consequences in wildlife, which can threaten the survival of species or populations.

An important limitation of the study is that factors affecting the stress-related hormone concentrations of otters could have been more extensive. For example, Majelantle et al. (2021) found that otters occurring in transformed areas occur in higher densities compared to natural areas, which could have been a contributing factor to otter stress-related hormone levels. Thus, other environmental and physiological factors not assessed in this study such as reproductive physiology, population density, habitat productivity etc. should be considered in future studies. Additionally, our sample size did not allow for comparison between sex and diet but could provide a more comprehensive understanding of potential ecological or behavioural differences that could affect fGCM levels in males and females.

### Conclusion

Results from this study highlight that the consumption of an anthropogenically provided resource such as trout is a major driver of glucocorticoid secretion. The long-term effects associated with a homogenous, anthropogenically supplied resource can result in obesity and increased parasite loads. Chronic adrenocortical response can influence animal behaviour, responses to environmental and anthropogenic disturbance as well as reproductive success. Our findings emphasize the importance of maintaining intact aquatic systems that support a naturally varied prey base for African clawless otter conservation. Future research should include in-depth dietary analysis at multiple transformed sites to assess and compare the influence of anthropogenically provided food sources on body mass, parasite load and glucocorticoid secretion. Collaborations with water quality, aquatic diversity and land use specialists, information can be used to identify specific factors that influence adrenocortical function and subsequent conservation success of African clawless otters at transformed sites. Information can further be used to promote actionable policy that maintains aquatic environment diversity and integrity, while supporting commercial ventures.

## Supplementary Material

Web_Material_coaf087

## Data Availability

Data is available at https://doi.org/10.25403/UPresearchdata.24324880.v1.
