# Supplementary figures and images for: Diet and traffic: anthropogenic factors that influence stress-related hormone levels in African clawless otters
Source: Conserv Physiol. 2025 Dec 13;13(1):coaf087. doi: 10.1093/conphys/coaf087 (PMC12703489; doi:10.1093/conphys/coaf087)

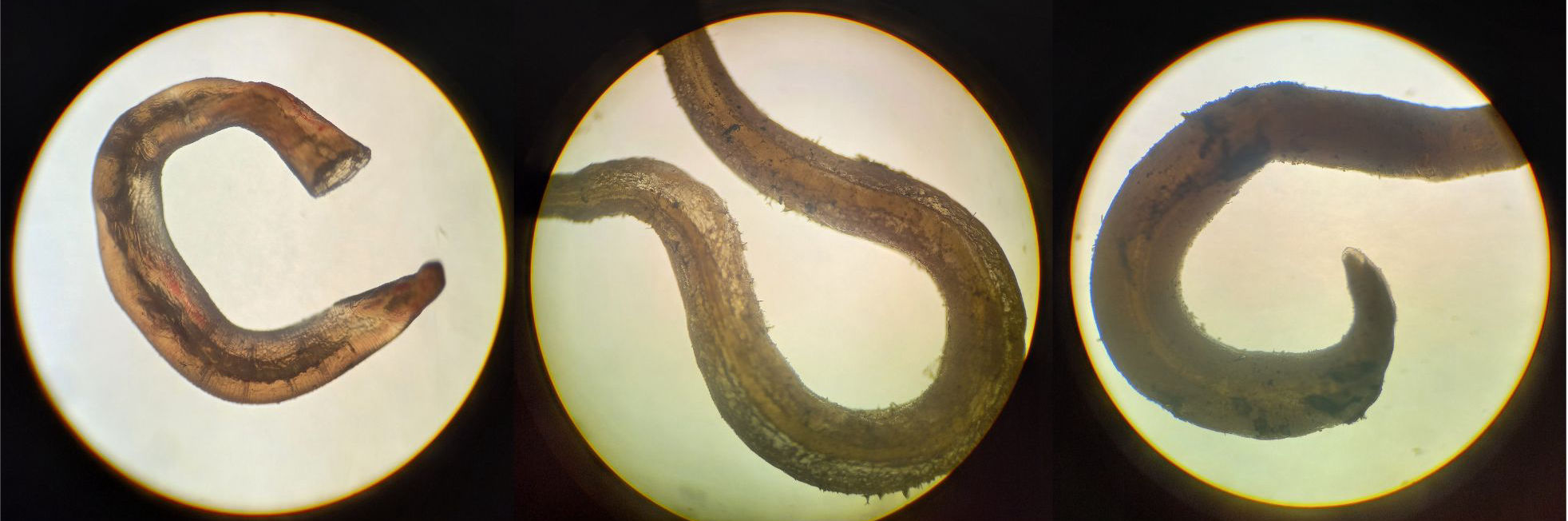

Supplement: Web_Material_coaf087 [file web_material_coaf087.zip › Figure S1.jpg]
